# Diethyl 1-benzyl-2,2-dioxo-4-phenyl-3,4,6,7,8,8a-hexa­hydro-1*H*-pyrrolo­[2,1-*c*][1,4]thia­zine-1,3-dicarboxyl­ate

**DOI:** 10.1107/S1600536811031047

**Published:** 2011-08-06

**Authors:** A. Chitradevi, S. Athimoolam, S. Asath Bahadur, S. Indumathi, S. Perumal

**Affiliations:** a Department of Physics, Sri Subramanya College of Engineering & Technology, Palani 624 615, India; b Department of Physics, University College of Engineering Nagercoil, Anna University of Technology Tirunelveli, Nagercoil 629 004, India; c Department of Physics, Kalasalingam University, Krishnan koil 626 190, India; d Department of Organic Chemistry, Madurai Kamaraj University, Madurai 625 021, India

## Abstract

In the title compound, C_26_H_31_NO_6_S, the five-membered pyrrolidine ring adopts an envelope conformation and the six-membered thia­zine ring is in a distorted chair conformation. The crystal packing is stabilized through an inter­molecular C—H⋯O inter­action, generating inversion-related *R*_2_^2^(10) ring motifs.

## Related literature

For the biological and pharmacological importance of thia­zine compounds, see: Moriyama *et al.* (2004 ▶); Koketsu *et al.* (2002 ▶). For the biological and pharmacological properties of compounds containing the pyrrolidine sub-structure, see: Hemming & Patel (2004 ▶); Kueh *et al.* (2003 ▶). For biological properties of compounds containing the pyrrolo­thia­zine scaffold, see: Armenise *et al.* (1991 ▶, 1998 ▶). For ring puckering analysis, see: Cremer & Pople (1975 ▶). For hydrogen-bonding inter­actions, see: Desiraju & Steiner (1999 ▶). For graph-set analysis, see: Etter *et al.* (1990 ▶).
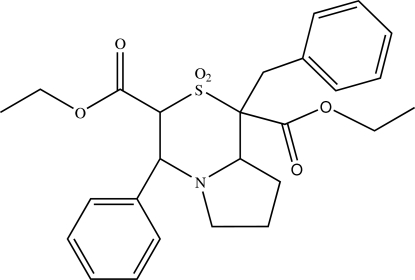


## Experimental

### 

#### Crystal data

C_26_H_31_NO_6_S
*M* *_r_* = 485.58
Monoclinic, 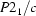

*a* = 13.5232 (9) Å
*b* = 16.8402 (12) Å
*c* = 12.1789 (9) Å
β = 116.568 (1)°
*V* = 2480.7 (3) Å^3^
*Z* = 4
Mo *K*α radiation
μ = 0.17 mm^−1^
*T* = 293 K
0.22 × 0.18 × 0.15 mm

#### Data collection

Bruker SMART APEX CCD area-detector diffractometer
23528 measured reflections
4360 independent reflections
3885 reflections with *I* > 2σ(*I*)
*R*_int_ = 0.023

#### Refinement

*R*[*F* ^2^ > 2σ(*F* ^2^)] = 0.040
*wR*(*F* ^2^) = 0.112
*S* = 1.05
4360 reflections
328 parameters
H-atom parameters constrained
Δρ_max_ = 0.34 e Å^−3^
Δρ_min_ = −0.21 e Å^−3^

### 

Data collection: *SMART* (Bruker, 2007 ▶); cell refinement: *SAINT* (Bruker, 2007 ▶); data reduction: *SAINT*; program(s) used to solve structure: *SHELXS97* (Sheldrick, 2008 ▶); program(s) used to refine structure: *SHELXL97* (Sheldrick, 2008 ▶); molecular graphics: *PLATON* (Spek, 2009 ▶); software used to prepare material for publication: *SHELXTL/PC* (Sheldrick, 2008 ▶).

## Supplementary Material

Crystal structure: contains datablock(s) global, I. DOI: 10.1107/S1600536811031047/su2277sup1.cif

Structure factors: contains datablock(s) I. DOI: 10.1107/S1600536811031047/su2277Isup2.hkl

Supplementary material file. DOI: 10.1107/S1600536811031047/su2277Isup3.cml

Additional supplementary materials:  crystallographic information; 3D view; checkCIF report

## Figures and Tables

**Table 1:** Hydrogen-bond geometry (Å, °)

*D*—H⋯*A*	*D*—H	H⋯*A*	*D*⋯*A*	*D*—H⋯*A*
C5—H5⋯O11^i^	0.98	2.51	3.447 (2)	159

Symmetry code: (i) 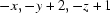.

**Fig. 1.**
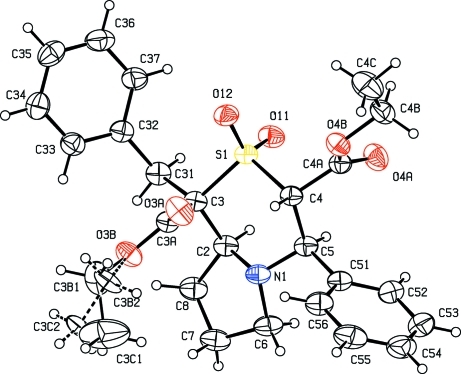
The molecular structure of the title molecule with atom numbering scheme and 30% probability displacement ellipsoids. H-bonds are shown as dashed lines.

**Fig. 2.**
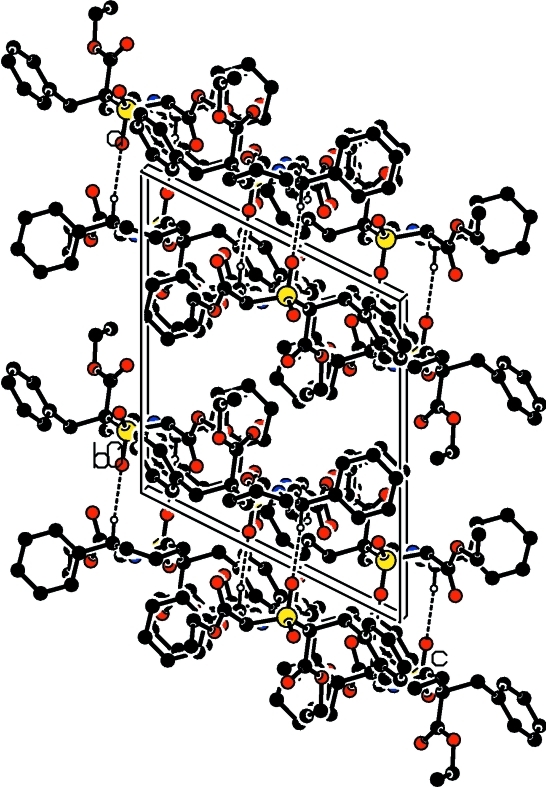
Packing diagram of the title compound viewed down the b-axis. H-bonds are shown as dashed lines (see Table 1 for details).

## supplementary crystallographic information

### Comment

Thiazines occupy a unique place in medicinal chemistry since they show diverse biological properties, such as antifungal, anti-inflammatory, anti-HIV, anti-psoriatic, sedative, neuroleptic, antitussive and anti-tubercular (Moriyama *et al.*, 2004; Koketsu *et al.*, 2002). In addition, compounds with a pyrrolidine sub-structure exhibit anti-tumour, analgesic, antidepressant, antihistaminic, anti-asthmatic and anti-Parkinson activities (Hemming & Patel, 2004; Kueh *et al.*, 2003). Compounds containing the pyrrolothiazine scaffold have also been shown to exhibit anti-inflammatory, anti-fungal and anti-microbial activities (Armenise *et al.*, 1998; Armenise *et al.*, 1991).

The molecular structure of the title molecule is illustrated in Fig. 1. The five-membered pyrrolidine ring has an envelope conformation [puckering parameters: Q(2) = 0.412 (2) Å, φ(2) = 152.9 (3)°; Cremer & Pople, 1975], with atom C6 at the flap. The six-membered thazine ring adopts a slightly distorted chair conformation [Puckering parameters: Q(2) = 0.1011 (19) Å, φ(2) = 101.2 (9) ° and Q(3) = 0.6610 (17) Å]. The dihedral angle between the phenyl rings is 54.3 (1)°. The planes of the carboxylate groups (CO_2_) are oriented with a dihedral angle of 22.5 (3)°.

In the crystal molecules are linked via intermolecular C—H···O interactions (Desiraju & Steiner, 1999). This interaction makes a *R*_2_^2^(10) ring motif centered about an inversion center (Table 1, Fig. 2; Etter *et al.*, 1990).

### Experimental

A mixture of ethyl 2-[(2-ethoxy-2-oxoethyl)sulfonyl]acetate (1.6 mmol), benzaldehyde (3.2 mmol) and pyrrolidine (1.6 mmol) was dissolved in ethanol (10 ml), heated until the solution turned yellow and stirred at room temperature for 2–5 days. After completion of the reaction, the crude product was purified using flash column chromatography on silica gel (230–400 mesh) with petroleum ether and ethyl acetate mixture (95:5 *v*/*v*) as an eluent. Crystals, suitable for X-ray diffraction analysis, were obtained by recrystallization from ethanol.

### Refinement

All the H atoms were positioned geometrically and treated as rding atoms: C—H = 0.93, 0.98, 0.97 and 0.96 Å for CH(methine), CH(aromatic), CH_2_ and CH_3_ H-atoms, respectively, with U_iso_(H)= k × *U*_eq_(C), where k = 1.5 for CH_3_ H-atoms and k = 1.2 for all other H-atoms. One of the side chains, -CH_2_-CH_3_, is disordered over two positions. The site occupancies of theses atoms (C3B1,C3B2) and (C3C1,C3C2) were fixed at 0.6 and 0.4, respectively.

### Crystal data

**Table:**

C_26_H_31_NO_6_S	*F*(000) = 1032
*M**_r_* = 485.58	*D*_x_ = 1.300 Mg m^−^^3^
Monoclinic, *P*2_1_/*c*	Melting point: 419 K
Hall symbol: -P 2ybc	Mo *K*α radiation, λ = 0.71073 Å
*a* = 13.5232 (9) Å	Cell parameters from 3512 reflections
*b* = 16.8402 (12) Å	θ = 2.4–23.8°
*c* = 12.1789 (9) Å	µ = 0.17 mm^−^^1^
β = 116.568 (1)°	*T* = 293 K
*V* = 2480.7 (3) Å^3^	Block, colourless
*Z* = 4	0.22 × 0.18 × 0.15 mm

### Data collection

**Table:**

Bruker SMART APEX CCD area-detector diffractometer	3885 reflections with *I* > 2σ(*I*)
Radiation source: fine-focus sealed tube	*R*_int_ = 0.023
graphite	θ_max_ = 25.0°, θ_min_ = 1.7°
ω scans	*h* = −15→16
23528 measured reflections	*k* = −19→19
4360 independent reflections	*l* = −14→14

### Refinement

**Table:**

Refinement on *F*^2^	Primary atom site location: structure-invariant direct methods
Least-squares matrix: full	Secondary atom site location: difference Fourier map
*R*[*F*^2^ > 2σ(*F*^2^)] = 0.040	Hydrogen site location: inferred from neighbouring sites
*wR*(*F*^2^) = 0.112	H-atom parameters constrained
*S* = 1.05	*w* = 1/[σ^2^(*F*_o_^2^) + (0.0573*P*)^2^ + 0.6606*P*] where *P* = (*F*_o_^2^ + 2*F*_c_^2^)/3
4360 reflections	(Δ/σ)_max_ = 0.001
328 parameters	Δρ_max_ = 0.34 e Å^−^^3^
0 restraints	Δρ_min_ = −0.21 e Å^−^^3^

### Special details

**Table:**

Geometry. Bond distances, angles etc. have been calculated using the rounded fractional coordinates. All su's are estimated from the variances of the (full) variance-covariance matrix. The cell esds are taken into account in the estimation of distances, angles and torsion angles
Refinement. Refinement of *F*^2^ against ALL reflections. The weighted *R*-factor *wR* and goodness of fit *S* are based on *F*^2^, conventional *R*-factors *R* are based on *F*, with *F* set to zero for negative *F*^2^. The threshold expression of *F*^2^ > σ(*F*^2^) is used only for calculating *R*-factors(gt) *etc*. and is not relevant to the choice of reflections for refinement. *R*-factors based on *F*^2^ are statistically about twice as large as those based on *F*, and *R*- factors based on ALL data will be even larger.

### Fractional atomic coordinates and isotropic or equivalent isotropic displacement parameters (Å^2^)

**Table:**

	*x*	*y*	*z*	*U*_iso_*/*U*_eq_	Occ. (<1)
S1	0.15741 (3)	0.92139 (2)	0.43420 (4)	0.0492 (1)	
O3A	0.35985 (10)	0.97564 (9)	0.43234 (14)	0.0719 (5)	
O3B	0.29527 (10)	1.07744 (8)	0.30412 (13)	0.0659 (5)	
O4A	0.15678 (12)	0.88688 (9)	0.70359 (14)	0.0746 (5)	
O4B	0.31214 (10)	0.83490 (7)	0.71181 (12)	0.0603 (4)	
O11	0.04484 (10)	0.91684 (8)	0.41508 (13)	0.0635 (5)	
O12	0.20400 (11)	0.85244 (7)	0.40719 (12)	0.0621 (4)	
N1	0.22020 (11)	1.09127 (8)	0.53463 (13)	0.0514 (4)	
C3B1	0.4011 (11)	1.0955 (8)	0.3087 (12)	0.091 (4)	0.600
C2	0.13365 (14)	1.07966 (10)	0.40838 (16)	0.0518 (5)	
C3C1	0.4416 (7)	1.1711 (6)	0.3650 (10)	0.177 (5)	0.600
C3	0.16594 (12)	1.00898 (9)	0.34934 (15)	0.0475 (5)	
C3A	0.28510 (13)	1.01706 (10)	0.36781 (16)	0.0505 (5)	
C4	0.24346 (13)	0.94980 (9)	0.59187 (15)	0.0476 (5)	
C4A	0.23023 (14)	0.88740 (10)	0.67482 (16)	0.0524 (5)	
C4B	0.30779 (17)	0.77085 (12)	0.79068 (19)	0.0686 (7)	
C4C	0.3898 (2)	0.71084 (15)	0.7988 (3)	0.0970 (10)	
C5	0.21555 (13)	1.03327 (10)	0.62232 (16)	0.0505 (5)	
C6	0.20688 (18)	1.17373 (11)	0.5649 (2)	0.0677 (7)	
C7	0.19111 (19)	1.21696 (11)	0.4504 (2)	0.0739 (7)	
C8	0.11953 (17)	1.16161 (11)	0.34662 (19)	0.0662 (7)	
C31	0.07930 (13)	1.00024 (11)	0.21296 (16)	0.0562 (6)	
C32	0.09916 (13)	0.93866 (10)	0.13463 (16)	0.0524 (5)	
C33	0.16040 (15)	0.95665 (12)	0.07280 (17)	0.0624 (6)	
C34	0.17452 (18)	0.90259 (14)	−0.00374 (19)	0.0717 (8)	
C35	0.12652 (19)	0.82867 (13)	−0.02086 (19)	0.0738 (7)	
C36	0.0654 (2)	0.80987 (13)	0.0397 (2)	0.0793 (8)	
C37	0.05118 (16)	0.86437 (12)	0.11638 (19)	0.0683 (7)	
C51	0.29799 (15)	1.05548 (10)	0.75148 (17)	0.0546 (6)	
C52	0.2635 (2)	1.06714 (12)	0.8413 (2)	0.0710 (8)	
C53	0.3373 (2)	1.08980 (14)	0.9581 (2)	0.0883 (10)	
C54	0.4456 (2)	1.10084 (14)	0.9869 (2)	0.0895 (9)	
C55	0.48211 (19)	1.09012 (14)	0.8991 (2)	0.0857 (9)	
C56	0.40837 (16)	1.06721 (12)	0.7808 (2)	0.0683 (7)	
C3B2	0.4149 (13)	1.0937 (6)	0.3395 (18)	0.062 (4)	0.400
C3C2	0.4129 (7)	1.1751 (5)	0.2913 (6)	0.069 (3)	0.400
H3B2	0.39440	1.09570	0.22600	0.1100*	0.600
H3C4	0.39340	1.21230	0.31500	0.2650*	0.600
H4	0.32070	0.94970	0.60600	0.0570*	
H3C5	0.51470	1.17950	0.37260	0.2650*	0.600
H5	0.14100	1.03280	0.61670	0.0610*	
H3C6	0.44390	1.17220	0.44490	0.2650*	0.600
H6A	0.27210	1.19240	0.63570	0.0810*	
H6B	0.14290	1.17950	0.58040	0.0810*	
H7A	0.26160	1.22640	0.44960	0.0890*	
H7B	0.15430	1.26740	0.44380	0.0890*	
H8A	0.14450	1.16030	0.28330	0.0790*	
H8B	0.04280	1.17830	0.31020	0.0790*	
H4B1	0.23440	0.74760	0.75600	0.0820*	
H4B2	0.32490	0.79120	0.87170	0.0820*	
H4C1	0.37360	0.69250	0.71780	0.1450*	
H4C2	0.38700	0.66690	0.84750	0.1450*	
H4C3	0.46240	0.73390	0.83620	0.1450*	
H31A	0.00850	0.98860	0.21160	0.0670*	
H31B	0.07240	1.05140	0.17370	0.0670*	
H33	0.19290	1.00650	0.08310	0.0750*	
H34	0.21660	0.91600	−0.04400	0.0860*	
H35	0.13550	0.79200	−0.07280	0.0890*	
H36	0.03310	0.75990	0.02910	0.0950*	
H37	0.00880	0.85080	0.15620	0.0820*	
H52	0.18960	1.05960	0.82260	0.0850*	
H53	0.31280	1.09760	1.01750	0.1060*	
H54	0.49520	1.11570	1.06620	0.1070*	
H55	0.55620	1.09820	0.91880	0.1030*	
H56	0.43310	1.05980	0.72150	0.0820*	
H3B1	0.45370	1.05450	0.35490	0.1100*	0.600
H2	0.06460	1.06630	0.41180	0.0620*	
H3B3	0.45890	1.09190	0.42780	0.0740*	0.400
H3B4	0.44420	1.05570	0.30180	0.0740*	0.400
H3C1	0.36030	1.17710	0.20620	0.1030*	0.400
H3C2	0.48500	1.18830	0.29970	0.1030*	0.400
H3C3	0.39210	1.21240	0.33690	0.1030*	0.400

### Atomic displacement parameters (Å^2^)

**Table:**

	*U*^11^	*U*^22^	*U*^33^	*U*^12^	*U*^13^	*U*^23^
S1	0.0454 (2)	0.0417 (2)	0.0580 (3)	−0.0019 (2)	0.0209 (2)	−0.0040 (2)
O3A	0.0435 (7)	0.0786 (9)	0.0905 (10)	0.0157 (6)	0.0273 (7)	0.0259 (8)
O3B	0.0477 (7)	0.0663 (8)	0.0768 (9)	−0.0028 (6)	0.0218 (6)	0.0149 (7)
O4A	0.0731 (9)	0.0739 (9)	0.0948 (10)	0.0031 (7)	0.0537 (8)	0.0090 (8)
O4B	0.0588 (7)	0.0533 (7)	0.0682 (8)	0.0028 (6)	0.0279 (6)	0.0108 (6)
O11	0.0481 (7)	0.0620 (8)	0.0773 (9)	−0.0112 (6)	0.0252 (6)	−0.0060 (6)
O12	0.0750 (8)	0.0440 (6)	0.0674 (8)	0.0058 (6)	0.0320 (7)	−0.0032 (6)
N1	0.0450 (7)	0.0401 (7)	0.0608 (8)	0.0022 (6)	0.0163 (6)	−0.0026 (6)
C3B1	0.059 (5)	0.136 (8)	0.073 (5)	−0.009 (4)	0.024 (4)	0.012 (4)
C2	0.0408 (8)	0.0450 (9)	0.0628 (10)	0.0054 (7)	0.0172 (8)	0.0000 (7)
C3C1	0.087 (5)	0.147 (7)	0.299 (13)	−0.048 (5)	0.089 (8)	−0.088 (9)
C3	0.0389 (8)	0.0423 (8)	0.0561 (10)	0.0023 (6)	0.0165 (7)	0.0012 (7)
C3A	0.0429 (9)	0.0500 (9)	0.0533 (9)	0.0002 (7)	0.0169 (7)	0.0006 (7)
C4	0.0411 (8)	0.0438 (9)	0.0560 (9)	−0.0004 (7)	0.0200 (7)	−0.0023 (7)
C4A	0.0516 (9)	0.0478 (9)	0.0575 (10)	−0.0047 (7)	0.0241 (8)	−0.0052 (8)
C4B	0.0715 (12)	0.0615 (12)	0.0663 (12)	−0.0063 (10)	0.0249 (10)	0.0130 (9)
C4C	0.1060 (19)	0.0645 (14)	0.119 (2)	0.0164 (13)	0.0489 (16)	0.0299 (14)
C5	0.0422 (8)	0.0453 (9)	0.0622 (10)	0.0011 (7)	0.0218 (8)	−0.0054 (7)
C6	0.0679 (12)	0.0431 (10)	0.0787 (13)	0.0041 (8)	0.0207 (10)	−0.0070 (9)
C7	0.0739 (13)	0.0438 (10)	0.0942 (15)	0.0042 (9)	0.0289 (11)	0.0022 (10)
C8	0.0622 (11)	0.0486 (10)	0.0777 (13)	0.0125 (8)	0.0224 (10)	0.0064 (9)
C31	0.0405 (9)	0.0590 (10)	0.0583 (10)	0.0040 (7)	0.0124 (8)	0.0015 (8)
C32	0.0403 (8)	0.0545 (10)	0.0495 (9)	0.0000 (7)	0.0085 (7)	0.0015 (7)
C33	0.0599 (11)	0.0599 (11)	0.0597 (11)	−0.0040 (9)	0.0199 (9)	0.0026 (9)
C34	0.0684 (13)	0.0833 (15)	0.0618 (12)	0.0042 (11)	0.0278 (10)	0.0008 (11)
C35	0.0768 (14)	0.0715 (13)	0.0579 (11)	0.0145 (11)	0.0165 (10)	−0.0062 (10)
C36	0.0844 (15)	0.0594 (12)	0.0753 (14)	−0.0124 (11)	0.0190 (12)	−0.0104 (10)
C37	0.0623 (11)	0.0684 (12)	0.0673 (12)	−0.0166 (10)	0.0228 (10)	−0.0087 (10)
C51	0.0536 (10)	0.0427 (9)	0.0619 (11)	0.0002 (7)	0.0208 (8)	−0.0037 (8)
C52	0.0789 (14)	0.0624 (12)	0.0734 (13)	−0.0089 (10)	0.0355 (11)	−0.0122 (10)
C53	0.118 (2)	0.0741 (15)	0.0689 (14)	−0.0171 (14)	0.0382 (14)	−0.0137 (11)
C54	0.109 (2)	0.0680 (14)	0.0611 (13)	−0.0189 (13)	0.0109 (13)	−0.0026 (11)
C55	0.0618 (13)	0.0702 (14)	0.0918 (17)	−0.0102 (10)	0.0045 (12)	0.0033 (12)
C56	0.0539 (11)	0.0655 (12)	0.0722 (13)	−0.0026 (9)	0.0164 (9)	−0.0039 (10)
C3B2	0.036 (4)	0.050 (4)	0.089 (9)	−0.003 (3)	0.018 (5)	0.030 (4)
C3C2	0.074 (5)	0.054 (4)	0.083 (4)	−0.019 (3)	0.040 (4)	0.014 (3)

### Geometric parameters (Å, °)

**Table:**

S1—O11	1.4354 (16)	C55—C56	1.391 (3)
S1—O12	1.4279 (14)	C3B1—H3B1	0.9700
S1—C3	1.8341 (16)	C3B1—H3B2	0.9700
S1—C4	1.8087 (17)	C2—H2	0.9800
O3A—C3A	1.190 (2)	C3C1—H3C4	0.9600
O3B—C3B1	1.440 (16)	C3C1—H3C5	0.9600
O3B—C3A	1.323 (2)	C3C1—H3C6	0.9600
O3B—C3B2	1.50 (2)	C3B2—H3B3	0.9700
O4A—C4A	1.192 (3)	C3B2—H3B4	0.9700
O4B—C4A	1.329 (2)	C4—H4	0.9800
O4B—C4B	1.463 (2)	C3C2—H3C1	0.9600
N1—C2	1.471 (2)	C3C2—H3C3	0.9600
N1—C5	1.469 (2)	C3C2—H3C2	0.9600
N1—C6	1.468 (2)	C4B—H4B2	0.9700
C3B1—C3C1	1.434 (17)	C4B—H4B1	0.9700
C2—C3	1.551 (2)	C4C—H4C2	0.9600
C2—C8	1.542 (3)	C4C—H4C3	0.9600
C3—C3A	1.530 (3)	C4C—H4C1	0.9600
C3—C31	1.553 (2)	C5—H5	0.9800
C3B2—C3C2	1.487 (15)	C6—H6B	0.9700
C4—C4A	1.523 (2)	C6—H6A	0.9700
C4—C5	1.543 (2)	C7—H7A	0.9700
C4B—C4C	1.471 (4)	C7—H7B	0.9700
C5—C51	1.513 (3)	C8—H8A	0.9700
C6—C7	1.501 (3)	C8—H8B	0.9700
C7—C8	1.520 (3)	C31—H31B	0.9700
C31—C32	1.512 (3)	C31—H31A	0.9700
C32—C33	1.379 (3)	C33—H33	0.9300
C32—C37	1.381 (3)	C34—H34	0.9300
C33—C34	1.376 (3)	C35—H35	0.9300
C34—C35	1.376 (3)	C36—H36	0.9300
C35—C36	1.369 (4)	C37—H37	0.9300
C36—C37	1.383 (3)	C52—H52	0.9300
C51—C56	1.385 (3)	C53—H53	0.9300
C51—C52	1.382 (3)	C54—H54	0.9300
C52—C53	1.377 (3)	C55—H55	0.9300
C53—C54	1.358 (4)	C56—H56	0.9300
C54—C55	1.376 (4)		
			
O11—S1—O12	117.67 (9)	H3C5—C3C1—H3C6	110.00
O11—S1—C3	106.32 (8)	H3B3—C3B2—H3B4	109.00
O11—S1—C4	108.57 (9)	O3B—C3B2—H3B3	111.00
O12—S1—C3	112.26 (8)	O3B—C3B2—H3B4	111.00
O12—S1—C4	108.60 (8)	C3C2—C3B2—H3B3	111.00
C3—S1—C4	102.28 (7)	C3C2—C3B2—H3B4	111.00
C3B1—O3B—C3A	120.1 (5)	C5—C4—H4	108.00
C3B2—O3B—C3A	110.7 (6)	C4A—C4—H4	108.00
C4A—O4B—C4B	116.17 (16)	S1—C4—H4	108.00
C2—N1—C5	113.51 (14)	H3C1—C3C2—H3C3	110.00
C2—N1—C6	104.99 (14)	C3B2—C3C2—H3C1	109.00
C5—N1—C6	113.35 (15)	C3B2—C3C2—H3C2	109.00
O3B—C3B1—C3C1	111.5 (11)	H3C2—C3C2—H3C3	109.00
N1—C2—C3	109.23 (15)	H3C1—C3C2—H3C2	109.00
N1—C2—C8	104.95 (14)	C3B2—C3C2—H3C3	109.00
C3—C2—C8	117.37 (16)	C4C—C4B—H4B2	110.00
S1—C3—C2	104.73 (11)	H4B1—C4B—H4B2	109.00
S1—C3—C3A	108.43 (11)	C4C—C4B—H4B1	110.00
S1—C3—C31	108.60 (11)	O4B—C4B—H4B1	110.00
C2—C3—C3A	111.10 (14)	O4B—C4B—H4B2	110.00
C2—C3—C31	109.53 (14)	C4B—C4C—H4C2	110.00
C3A—C3—C31	114.00 (14)	C4B—C4C—H4C3	110.00
O3B—C3B2—C3C2	103.2 (11)	C4B—C4C—H4C1	109.00
O3A—C3A—O3B	123.89 (19)	H4C2—C4C—H4C3	109.00
O3A—C3A—C3	124.94 (16)	H4C1—C4C—H4C2	109.00
O3B—C3A—C3	111.16 (15)	H4C1—C4C—H4C3	109.00
S1—C4—C5	112.69 (12)	N1—C5—H5	109.00
C4A—C4—C5	110.81 (15)	C4—C5—H5	109.00
S1—C4—C4A	108.13 (11)	C51—C5—H5	109.00
O4A—C4A—O4B	124.99 (17)	N1—C6—H6A	111.00
O4A—C4A—C4	124.11 (17)	H6A—C6—H6B	109.00
O4B—C4A—C4	110.90 (17)	N1—C6—H6B	111.00
O4B—C4B—C4C	107.5 (2)	C7—C6—H6A	111.00
N1—C5—C4	109.65 (14)	C7—C6—H6B	111.00
C4—C5—C51	109.23 (14)	C6—C7—H7B	111.00
N1—C5—C51	109.89 (14)	C8—C7—H7A	111.00
N1—C6—C7	101.99 (16)	C8—C7—H7B	111.00
C6—C7—C8	104.37 (17)	C6—C7—H7A	111.00
C2—C8—C7	104.44 (16)	H7A—C7—H7B	109.00
C3—C31—C32	118.54 (15)	C2—C8—H8B	111.00
C31—C32—C33	120.88 (16)	C7—C8—H8A	111.00
C33—C32—C37	117.70 (18)	H8A—C8—H8B	109.00
C31—C32—C37	121.31 (18)	C7—C8—H8B	111.00
C32—C33—C34	121.6 (2)	C2—C8—H8A	111.00
C33—C34—C35	120.2 (2)	C3—C31—H31A	108.00
C34—C35—C36	119.0 (2)	H31A—C31—H31B	107.00
C35—C36—C37	120.7 (2)	C32—C31—H31B	108.00
C32—C37—C36	120.9 (2)	C3—C31—H31B	108.00
C5—C51—C52	120.4 (2)	C32—C31—H31A	108.00
C52—C51—C56	118.83 (19)	C34—C33—H33	119.00
C5—C51—C56	120.76 (18)	C32—C33—H33	119.00
C51—C52—C53	120.8 (3)	C35—C34—H34	120.00
C52—C53—C54	120.3 (2)	C33—C34—H34	120.00
C53—C54—C55	120.1 (2)	C34—C35—H35	120.00
C54—C55—C56	120.1 (2)	C36—C35—H35	120.00
C51—C56—C55	119.8 (2)	C37—C36—H36	120.00
O3B—C3B1—H3B1	109.00	C35—C36—H36	120.00
O3B—C3B1—H3B2	109.00	C32—C37—H37	120.00
C3C1—C3B1—H3B1	109.00	C36—C37—H37	120.00
C3C1—C3B1—H3B2	109.00	C51—C52—H52	120.00
H3B1—C3B1—H3B2	108.00	C53—C52—H52	120.00
N1—C2—H2	108.00	C52—C53—H53	120.00
C3—C2—H2	108.00	C54—C53—H53	120.00
C8—C2—H2	108.00	C55—C54—H54	120.00
C3B1—C3C1—H3C4	109.00	C53—C54—H54	120.00
C3B1—C3C1—H3C5	109.00	C56—C55—H55	120.00
C3B1—C3C1—H3C6	110.00	C54—C55—H55	120.00
H3C4—C3C1—H3C5	109.00	C51—C56—H56	120.00
H3C4—C3C1—H3C6	110.00	C55—C56—H56	120.00
			
O11—S1—C3—C2	60.76 (13)	S1—C3—C3A—O3B	177.24 (12)
O11—S1—C3—C3A	179.45 (11)	C2—C3—C3A—O3A	110.7 (2)
O11—S1—C3—C31	−56.19 (14)	C2—C3—C3A—O3B	−68.19 (18)
O12—S1—C3—C2	−169.24 (12)	C31—C3—C3A—O3A	−125.01 (19)
O12—S1—C3—C3A	−50.56 (13)	C31—C3—C3A—O3B	56.15 (19)
O12—S1—C3—C31	73.80 (14)	S1—C3—C31—C32	−70.74 (18)
C4—S1—C3—C2	−53.02 (13)	C2—C3—C31—C32	175.43 (15)
C4—S1—C3—C3A	65.66 (13)	C3A—C3—C31—C32	50.3 (2)
C4—S1—C3—C31	−169.97 (12)	S1—C4—C4A—O4A	−83.5 (2)
O11—S1—C4—C4A	59.37 (14)	S1—C4—C4A—O4B	97.50 (15)
O11—S1—C4—C5	−63.46 (14)	C5—C4—C4A—O4A	40.5 (2)
O12—S1—C4—C4A	−69.69 (15)	C5—C4—C4A—O4B	−138.54 (15)
O12—S1—C4—C5	167.49 (13)	S1—C4—C5—N1	−54.12 (18)
C3—S1—C4—C4A	171.48 (13)	S1—C4—C5—C51	−174.60 (13)
C3—S1—C4—C5	48.66 (15)	C4A—C4—C5—N1	−175.43 (15)
C3A—O3B—C3B1—C3C1	−114.7 (9)	C4A—C4—C5—C51	64.1 (2)
C3B1—O3B—C3A—O3A	1.1 (7)	N1—C5—C51—C52	123.55 (18)
C3B1—O3B—C3A—C3	180.0 (6)	N1—C5—C51—C56	−54.2 (2)
C4B—O4B—C4A—O4A	2.0 (3)	C4—C5—C51—C52	−116.12 (19)
C4B—O4B—C4A—C4	−179.00 (14)	C4—C5—C51—C56	66.2 (2)
C4A—O4B—C4B—C4C	167.85 (18)	N1—C6—C7—C8	39.0 (2)
C5—N1—C2—C3	−77.18 (19)	C6—C7—C8—C2	−19.6 (2)
C5—N1—C2—C8	156.15 (16)	C3—C31—C32—C33	−87.6 (2)
C6—N1—C2—C3	158.50 (16)	C3—C31—C32—C37	96.5 (2)
C6—N1—C2—C8	31.8 (2)	C31—C32—C33—C34	−176.65 (19)
C2—N1—C5—C4	66.77 (19)	C37—C32—C33—C34	−0.6 (3)
C2—N1—C5—C51	−173.16 (15)	C31—C32—C37—C36	176.75 (19)
C6—N1—C5—C4	−173.57 (16)	C33—C32—C37—C36	0.7 (3)
C6—N1—C5—C51	−53.5 (2)	C32—C33—C34—C35	0.4 (3)
C2—N1—C6—C7	−44.3 (2)	C33—C34—C35—C36	−0.3 (3)
C5—N1—C6—C7	−168.71 (17)	C34—C35—C36—C37	0.5 (4)
N1—C2—C3—S1	67.82 (16)	C35—C36—C37—C32	−0.7 (3)
N1—C2—C3—C3A	−49.04 (18)	C5—C51—C52—C53	−177.94 (18)
N1—C2—C3—C31	−175.88 (14)	C56—C51—C52—C53	−0.2 (3)
C8—C2—C3—S1	−172.95 (15)	C5—C51—C56—C55	177.91 (18)
C8—C2—C3—C3A	70.2 (2)	C52—C51—C56—C55	0.2 (3)
C8—C2—C3—C31	−56.7 (2)	C51—C52—C53—C54	−0.2 (3)
N1—C2—C8—C7	−7.0 (2)	C52—C53—C54—C55	0.6 (4)
C3—C2—C8—C7	−128.48 (19)	C53—C54—C55—C56	−0.6 (4)
S1—C3—C3A—O3A	−3.9 (2)	C54—C55—C56—C51	0.2 (3)

### Hydrogen-bond geometry (Å, °)

**Table:**

*D*—H···*A*	*D*—H	H···*A*	*D*···*A*	*D*—H···*A*
C5—H5···O11^i^	0.98	2.51	3.447 (2)	159

Symmetry codes: (i) −*x*, −*y*+2, −*z*+1.
